# Chemokine CXCL13–CXCR5 signaling in neuroinflammation and pathogenesis of chronic pain and neurological diseases

**DOI:** 10.1186/s11658-024-00653-y

**Published:** 2024-10-29

**Authors:** Kaige Zheng, Muyan Chen, Xingjianyuan Xu, Peiyi Li, Chengyu Yin, Jie Wang, Boyi Liu

**Affiliations:** 1 Department of Neurobiology and Acupuncture Research, The Third Clinical Medical College, Key Laboratory of Acupuncture and Neurology of Zhejiang Province, Zhejiang Chinese Medical University, Hangzhou, China; 2 Department of Rehabilitation in Traditional Chinese Medicine, The Second Affiliated Hospital of Zhejiang University School of Medicine, Hangzhou, China

**Keywords:** Chemokine, Neuroinflammation, Pain, Astrocyte, Neuron, CXCL13

## Abstract

Chronic pain dramatically affects life qualities of the sufferers. It has posed a heavy burden to both patients and the health care system. However, the current treatments for chronic pain are usually insufficient and cause many unwanted side effects. Chemokine C–X–C motif ligand 13 (CXCL13), formerly recognized as a B cell chemokine, binds with the cognate receptor CXCR5, a G-protein-coupled receptor (GPCR), to participate in immune cell recruitments and immune modulations. Recent studies further demonstrated that CXCL13–CXCR5 signaling is implicated in chronic pain via promoting neuroimmune interaction and neuroinflammation in the sensory system. In addition, some latest work also pointed out the involvement of CXCL13–CXCR5 in the pathogenesis of certain neurological diseases, including ischemic stroke and amyotrophic lateral sclerosis. Therefore, we aim to outline the recent findings in regard to the involvement of CXCL13–CXCR5 signaling in chronic pain as well as certain neurological diseases, with the focus on how this chemokine signaling contributes to the pathogenesis of these neurological diseases via regulating neuroimmune interaction and neuroinflammation. Strategies that can specifically target CXCL13–CXCR5 signaling in distinct locations may provide new therapeutic options for these neurological diseases.

## Introduction

Chronic pain is a debilitating condition that dramatically affects the life quality of suffering patients. According to epidemiology estimation, around 20% of the world’s population are suffering from chronic pain [1]. Chronic pain oftentimes lasts over months or even years that causes great amount of health cost and poses heavy financial burden to both the patients and the society [2]. Even worse, people who suffer from chronic pain are more prone to develop negative emotions, including anxiety, depression, and loss of motivation [3, 4]. Severe and long-lasting pain can even trigger suicidal tendencies among the sufferers [5]. Thus, chronic pain tortures the sufferers both physically and mentally. However, current treatment regimen for chronic pain, including nonsteroidal anti-inflammatory drugs (NSAIDs), opioids, and antidepressants, are usually insufficient and can cause certain adverse effects, including gastric ulcer, kidney damage, and addiction [6–8]. Therefore, to improve chronic pain management and reduce side effects, it is better to understand the mechanisms underlying chronic pain and identify novel therapeutic targets.

The immune system works together with the nervous system to contribute to neuroinflammation [9]. The neuroimmune interaction incorporates a number of signaling and types of cells, which include neurons, glia, immune cells, and other non-neuronal cells [8, 9]. It has become more and more evident that neuroinflammation plays a critical role in mediating and sustaining chronic pain and neurodegenerative diseases [10–15]. Neuroinflammation, which is mainly triggered by oxidative stress, inflammatory mediators, proinflammatory cytokines, or chemokines, can take place in local inflamed tissues, peripheral sensory nerves, spinal cord, or the brain during chronic pain [16–23]. Glial or immune cells contribute to neuroinflammation via releasing proinflammatory cytokines or chemokines [24, 25]. Conversely, these proinflammatory substances are produced from nerve endings and sensory neurons as well to trigger glia activation and immune cell infiltration [26]. Chemokines play an important role in the immune system by exerting chemotactic effect [27]. Recent evidence indicates a critical role of chemokine chemokine C–X–C motif ligand (CXCL)13–CXCR5 signaling in chronic pain and certain neurological diseases via mediating neuro-immune interaction and triggering neuroinflammation.

## Overview of CXCL13–CXCR5 signaling

The chemokine CXCL13 was initially termed as B lymphocyte chemoattractant (BLC) or B cell attracting chemokine 1 (BCA-1) due to the fact that it was the first chemokine found to exert selective chemotaxis on B cells [28, 29]. While CXCL13 is mainly chemotactic for B cell, it also attracts certain subtypes of T cells as well as macrophages [28, 30]. CXCL13 has been found to be constitutively expressed in B cell-rich follicles of the secondary lymphoid organs, including spleen and lymph nodes. [31]. CXCR5, a G-protein-coupled receptor (GPCR), which was once known as Burkitt’s lymphoma receptor 1 (BLR1), is the only receptor identified for CXCL13 so far. It is mainly distributed in B cells and shows expression in a subtype of CD4^+^ T cells and dendritic cells as well [31, 32]. CXCL13 binds with CXCR5 to produce chemotaxis or modulate cellular function of these types of cells to contribute to physiological functions including lymphoid neogenesis, lymphoid organization, and immune responses [33]. In addition to the expression on certain immune cells, emerging evidence also demonstrates that the peripheral and central nervous system has functional CXCR5 expression, including neurons and astrocytes [34, 35]. These findings, thus, provide a cellular basis for CXCL13–CXCL5 signaling to regulate chronic pain or neurological diseases via neuroimmune interaction. Under certain chronic pain and neurological disease conditions, CXCL13–CXCR5 expression or downstream signaling in the nervous system can be dysregulated, thus contributing to the pathogenesis of these diseases.

**Fig. 1:**
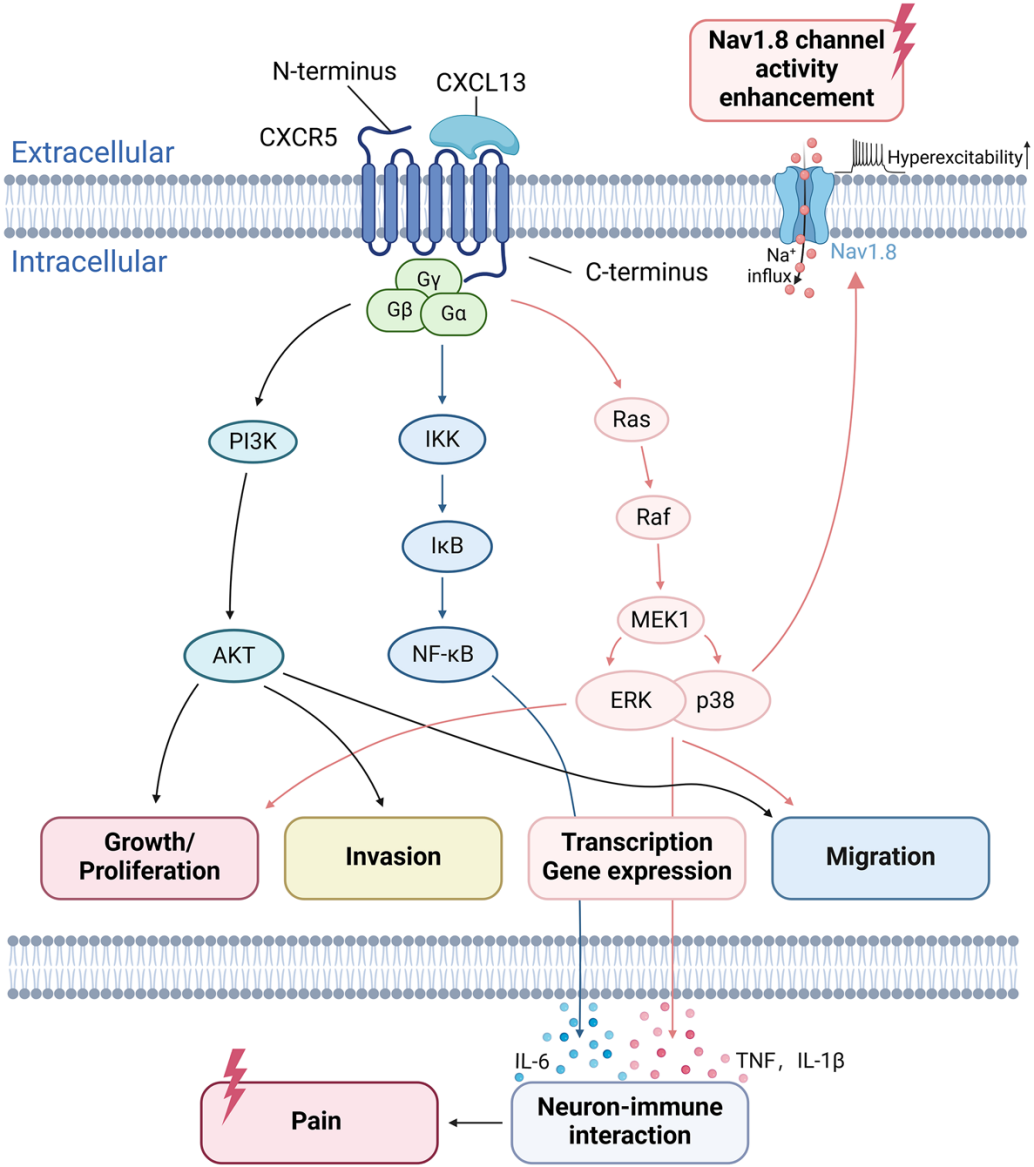
Intracellular signaling pathways conveyed by CXCL13–CXCR5. Under specific physiological or pathophysiological conditions, CXCL13 is released and acts on its receptor CXCR5, a GPCR, to activate distinct downstream signaling. These signaling pathways contribute to physiological functions or pathophysiological conditions including growth/proliferation, invasion, gene transcription, and migration. When tissue inflammation or nerve injury occurs, CXCL13–CXCR5-mediated gene transcription produces proinflammatory cytokines, including IL-1β, tumor necrosis factor (TNF), and IL-6, that are released into extracellular space. These cytokines can activate neurons or cause microglial activation via a neuroimmune interaction, contributing to the development and maintenance of chronic pain [34–36]. p38 MAPK activation promotes Nav1.8 channel activity in nociceptive neurons, resulting in neuron hyperexcitability and pain [37]. The schematic picture was created with BioRender

The intracellular signaling conveyed by CXCR5 is in accordance with classical GPCR activation mechanism, which consequently activates a number of intracellular signaling molecules, including nuclear factor-κB (NF-κB), protein kinase B (Akt), extracellular signal-regulated kinase (ERK), and p38 mitogen-activated protein kinase (p38 MAPK) (Fig. 1) [33]. It should be noted that the exact downstream signaling conveyed by CXCL13–CXCR5 may depend on types of cells and distinct physiology or pathophysiology conditions. After tissue inflammation or nerve injury occurs, the activation of these intracellular signaling conveyed through CXCL13–CXCR5, including NF-κB, ERK, and p38 MAPK, results in proinflammatory cytokine production that contributes to neuroimmune interaction and neuroinflammation, which is important for the development and maintenance of chronic pain (Fig. 1) [34, 36]. Moreover, CXCL13–CXCR5-mediated p38 MAPK activation can promote Nav1.8 channel activity, resulting in hyperexcitability of nociceptive sensory neurons (Fig. 1) [37]. Here, in this review, we aim to outline the recent findings in regard to the involvement of CXCL13–CXCR5 signaling in chronic pain as well as certain neurological diseases, with the focus on how this chemokine signaling regulates neuroimmune interaction and neuroinflammation to contribute to pathogenesis of these diseases.

## CXCL13 signaling in chronic pain

### CXCL13 in spinal nerve ligation (SNL)-induced neuropathic pain and affective disorder

**Fig. 2:**
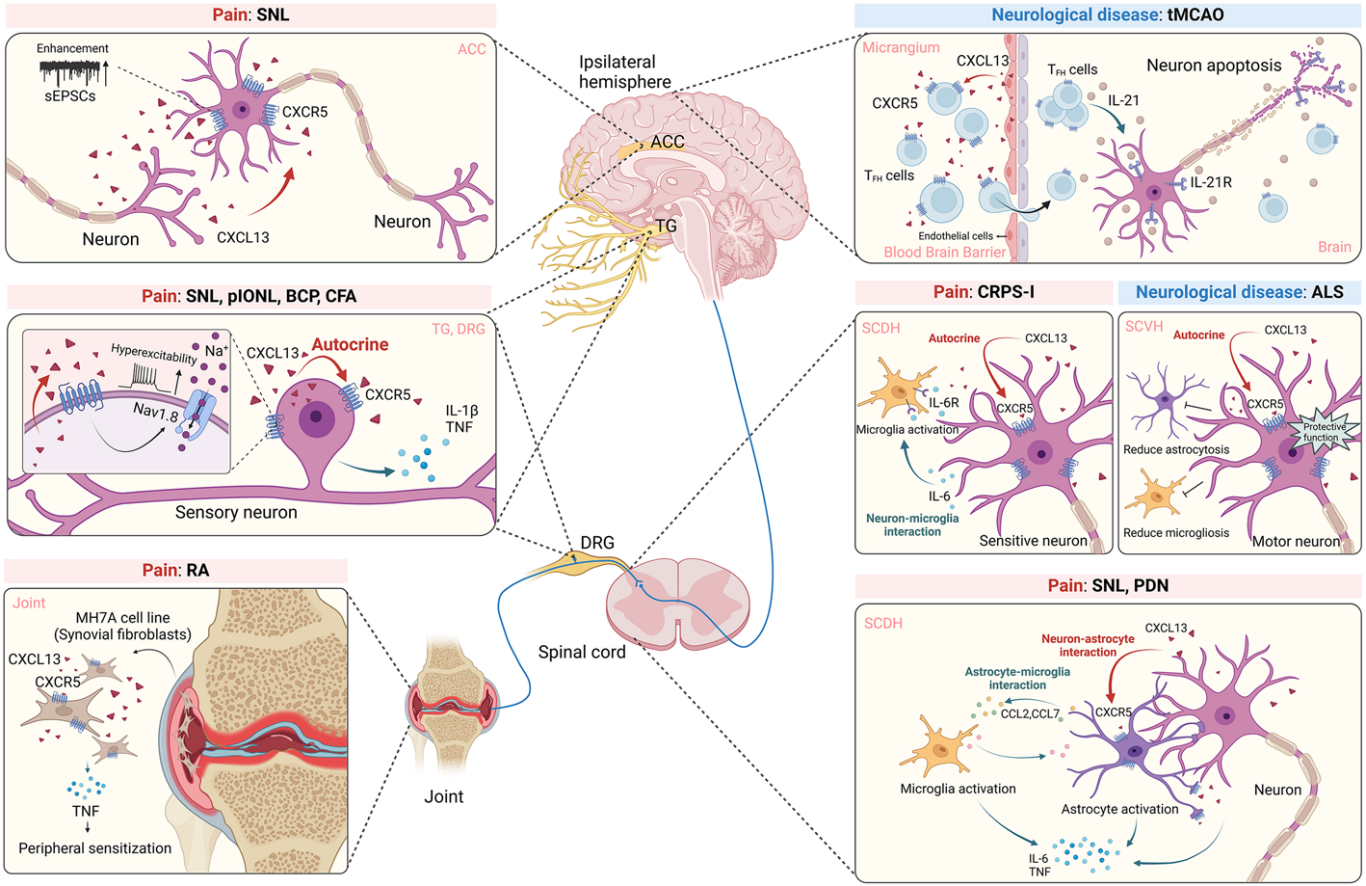
Summary of the neuroimmune interaction mediated via CXCL13–CXCR5 signaling in the nervous system during chronic pain and neurological diseases. In the periphery (joint), during RA, CXCL13 activates ERK and p38 MAPK via CXCR5 to promote TNF production in MH7A cells, which may contribute to joint inflammation and pain in RA [89]. In dorsal root ganglia (DRG) and TG, during chronic pain, CXCL13 is produced from sensory neurons and activates CXCR5 expressed by sensory neurons via an autocrine manner [36, 37]. CXCR5 activation promotes TNF and IL-1β production in sensory neurons and enhances Nav1.8 channel activity via p38 MAPK to promote nociceptive neuron hyperexcitability [36, 37]. In SCDH, during chronic pain, CXCL13 is produced from spinal neurons. On one hand, CXCL13 acts on CXCR5 expressed by astrocytes to promote astrocyte activation, resulting in proinflammatory cytokine production, such as CCL2 and CCL7 [35]. On the other hand, CXCL13 activates CXCR5 expressed by neurons via an autocrine manner to promote IL-6 production [34]. These cytokines either directly activate spinal neurons or cause microglial activation to augment and maintain chronic pain condition. Conversely, in spinal cord ventral horn (SCVH), CXCL13 is produced from spinal motor neurons and acts on CXCR5 expressed by motor neurons via an autocrine manner to exert protective effects on motor neuron loss by ameliorating astrocytosis and microgliosis during amyotrophic lateral sclerosis (ALS) [104]. In anterior cingulate cortex (ACC), during chronic pain, CXCL13 and CXCR5 are both expressed in neurons. CXCL13 increases spontaneous excitatory postsynaptic currents (sEPSCs) in ACC and CXCR5 contributes to the increases in glutamatergic synaptic transmission [44]. In ipsilateral hemisphere, during ischemic stroke, CXCL13 is expressed on the inflamed cerebral vessels and recruits IL-21-producing T follicular helper (T_FH_) cells via activating CXCR5 expressed by these cells. IL-21 released from infiltrated T_FH_ cells then activates interleukin-21 receptor (IL-21R) expressed on neurons to trigger neuronal death via Janus kinase (JAK)/STAT signaling [107]. The schematic picture was created with BioRender

The pioneering work from Jiang et al. identified a significant upregulation of CXCL13 expression in spinal cord dorsal horn (SCDH) in SNL model mice [35]. Immunostaining further identified that CXCL13 was mainly expressed by spinal neurons, whereas CXCR5 showed expression exclusively in spinal astrocytes and only partially in neurons in SNL model mice (Fig. 2, Table 1) [35]. *Cxcl13* gene knockdown in spinal cord attenuated neuropathic pain in SNL model mice [35]. The authors further identified a suppressive effect conducted by miR-186-5p, a microRNA colocalized with CXCL13, on CXCL13 expression in spinal neurons. Employing *Cxcr5* gene knockout strategy or conducting *Cxcr5* gene knockdown in spinal cord both ameliorated neuropathic pain of model mice [35]. Upon release from neurons, CXCL13 acted on astrocytic CXCR5 to trigger ERK-dependent astrocyte activation, resulting in the release of proinflammatory cytokines. These substances either directly acted on spinal neurons or cause microglia activation to augment and maintain pain signal transduction [38]. Thus, this work is the first study to demonstrate the contribution of CXCL13–CXCR5 signaling to chronic pain via mediating spinal neuron-astrocyte crosstalk, which paved the way for subsequent related studies. Targeting CXCL13 via inducing miR-186-5p expression or blocking CXCR5 in spinal cord may be a potential therapeutic option for neuropathic pain management.

**Table 1:** Summary of cellular distribution of CXCL13–CXCR5 in different models of pain or neurological diseases

Tissue type	Models for pain or neurological diseases	Marker	Cell type	Colocalized with	References
Ipsilateral hemisphere	tMCAO	ICAM-1	ICAM-1^+^cell	CXCL13	[107]
		CD4	CD4^+^cell	CXCL13–CXCR5	
Anterior cingulate cortex	SNL	NeuN	Neuron	*Cxcl13*/CXCR5	[44]
Trigeminal ganglion	pIONL	β-III tubulin	Neuron	CXCL13–CXCR5	[36]
Spinal cord	SNL	NeuN	Neuron	CXCL13–CXCR5	[35]
		MAP2	Neuron	CXCL13	
		GFAP	Astrocyte	CXCR5	
	PDN	NeuN	Neuron	CXCL13–CXCR5	[48]
		GFAP	Astrocyte	CXCR5	
		CD11b	Microglia	CXCR5	
	CPIP	NeuN	Neuron	CXCL13–CXCR5	[34]
		GFAP	Astrocyte	CXCL13–CXCR5	
	ALS	Neurotrace	Motor neuron	CXCL13–CXCR5	[104]
		CD68	Microglia	CXCL13	
Sciatic nerves	ALS	NF200	Efferent motor axon	CXCL13	[104]
		S100β	Bundles of Schwann cell	CXCR5	
		Smi32	Peripheral axon	CXCL13–CXCR5	
Dorsal root ganglion	SNL	TUJ1	Neuron	CXCL13	[40]
	CFA	IB4	Nonpeptidergic C-nociceptor	CXCL13–CXCR5	[37]
		CGRP	Peptidergic C-nociceptor		
		NF200	A-fiber afferent		

Gene expression change and its modulation in sensory ganglia, such as dorsal root ganglia (DRG), plays an important role in the development of neuropathic pain conditions [7]. Epigenetic mechanisms have been increasingly reported to contribute to chronic pain modulation [39]. Recently, Ma et al. reported an epigenetic modulatory mechanism of CXCL13’s production from DRG neurons of SNL-induced neuropathic pain model mice [40]. The authors found that the expression of a transcription factor called zinc finger protein 382 (ZNF382) was persistently downregulated in the injured DRG neurons following SNL model establishment. The downregulation of ZNF382 resulted in its loss of binding with the silencer located in the distal upstream of *Cxcl13* gene promoter, which weakened the inhibitory effect from epigenetic modification of *Cxcl13* gene promoter via reducing transcriptional silencing complex formation. This epigenetic modulatory mechanism, in turn, resulted in transcriptional activation of the *Cxcl13* gene in injured DRG neurons and subsequent production of the CXCL13 protein. CXCL13 released from injured DRG neurons conversely acted upon CXCR5 expressed in DRG neurons per se via an autocrine manner to further promote ERK-dependent production of inflammatory mediators, resulting in chronic pain [40]. Thus, epigenetic modification of *Cxcl13* gene expression in peripheral sensory neurons represents a novel mechanism underlying chronic neuropathic pain.

Patients with chronic neuropathic pain usually develop emotional disorders, including aversion, depression, and anxiety, that dramatically affect life quality [41]. Up to date, there is mounting evidence suggesting an important contribution of the anterior cingulate cortex (ACC) in the brain to pain-related negative emotions [42, 43]. The work by Wu et al. found that the expression of CXCL13–CXCR5 in ACC was remarkably upregulated in SNL model mice [44]. CXCL13 and CXCR5 were predominantly expressed in neurons of ACC (Fig. 2, Table 1). The SNL model mice displayed obvious pain-related aversive behavior, which was significantly improved by *Cxcr5* gene knockdown in ACC. Electrophysiology recording further revealed that CXCL13 perfusion increased the frequency and amplitude of spontaneous excitatory postsynaptic currents (EPSCs) in ACC slices, whereas *Cxcr5* knockdown reduced glutamatergic synaptic transmission increases in ACC slices of SNL model mice. Therefore, this work demonstrates that the increase in CXCL13–CXCR5 expression in ACC is involved in neuropathic pain-associated aversive behavior.

### CXCL13 in painful diabetic neuropathy

Painful diabetic neuropathy (PDN) is a common neurological symptom accompanying patients with diabetes mellitus [45]. PDN is characterized with stinging/burning sensation, numbness, and or loss of sensation at the distal end of the lower extremities, imposing heavy burdens on patients’ life quality [46]. However, some currently approved medications for PDN management, including tricyclic antidepressants, serotonin, and norepinephrine reuptake inhibitors or anticonvulsants, cannot produce sufficient relieving effects on patients, rendering it a quite challenging neurological condition in clinic [47]. Therefore, it is of great interest to identify new targets for PDN.

Recently, Liu et al. identified a critical role of spinal CXCL13–CXCR5 signaling in mediating PDN in a mouse model of type 2 diabetes [48]. This group utilized db/db (leptin receptor mutant) strain mice that exhibit hyperglycemia, obesity, and persistent mechanical pain hypersensitivity similar with human type 2 diabetes [49]. They found that CXCL13–CXCR5 expression in spinal cord was significantly upregulated in db/db mice. Immunofluorescence indicated CXCL13 was exclusively located on spinal neurons, whereas CXCR5 was predominantly distributed on spinal astrocytes, with few expression on spinal neurons or microglia (Fig. 2, Table 1), a result similar with the previous study [35]. ERK, AKT, and signal transducer and activator of transcription 3 (STAT3) are important signaling molecules involved in neuroinflammation, and their activation by phosphorylation is highly correlated with central sensitization [50–52]. The authors found db/db mice showed increased expression of p-ERK, p-AKT, and p-STAT3, as well as glial cell activation, in the spinal cord. Pharmacological blocking of ERK and STAT signaling attenuated mechanical allodynia of db/db mice. Intrathecal CXCL13 injection to naive mice triggered mechanical allodynia as well as an upregulation of p-ERK, p-AKT, and p-STAT3 expression in spinal cord. These effects were all abolished in mice deficient in *Cxcr5* (*Cxcr5*^−/−^). Finally, knocking down *Cxcr5* gene in spinal cord attenuated mechanical allodynia of db/db mice [48]. Thus, this study reveals an important contribution of spinal CXCL13–CXCR5 signaling to mechanical allodynia of diabetic model mice. The exact downstream signaling of CXCL13–CXCR5 that leads to spinal glial cell overactivation and pain hypersensitivity of diabetes model mice is still awaiting further investigation.

### CXCL13 in complex regional pain syndrome type I

Complex regional pain syndrome type I (CRPS-I) oftentimes occurs secondary to an initial injury, including surgery, fracture, and ischemia. It usually involves the patient’s extremities and may spread to other body regions [53, 54]. CRPS-I can cause severe and long-lasting pain that affects the patients [55]. However, conventional treatments, e.g., steroids or NSAIDs, do not produce desirable relieving effects on CRPS-I [56]..

To facilitate mechanism investigation of CRPS-I, the chronic postischemic pain (CPIP) model, which recapitulates several key pathological features of human CRPS-I, was developed to mimic CRPS-I [57–59]. By means of this animal model of CRPS-I, our recent work identified a critical contribution of spinal CXCL13–CXCR5 signaling to both mechanical and cold allodynia via autocrine mechanism in model mice [34]. Work from us and others have revealed that spinal neuroinflammation plays a critical role in mediating glial cell overactivation, central sensitization, and pain hypersensitivity in CRPS-I model animals [8, 60–63]. To further screen potential molecules or signaling involved in spinal neuroinflammation, we employed RNA sequencing (RNA-seq) to look for possible candidates in ipsilateral spinal cord dorsal horn (SCDH) of CPIP model rats. Our work identified the CXCL13 gene among the most upregulated genes in CPIP model rats [64]. The expression of CXCL13 and CXCR5 was further confirmed to be upregulated in ipsilateral SCDH using a mouse model of CPIP [34]. CXCL13 and CXCR5 were predominantly expressed in spinal neurons of CPIP model mice (Fig. 2, Table 1). Neutralizing spinal CXCL13 or genetic deletion of *Cxcr5* (*Cxcr5*^−/−^) reduced mechanical/cold allodynia, as well as spinal glial cell overactivation and c-Fos activation in SCDH of CPIP model mice, demonstrating an important role of CXCL13–CXCR5 signaling in mediating pain response of model mice [34]. It is known that patients with CRPS-I can develop emotional disorders owing to chronic pain [65]. Our work further examined the emotional disorders of CPIP model mice and found that CPIP model mice exhibited aversive behavior, an indication of emotional disorders similar with patients with CRPS-I. Interestingly, the aversive behavior was significantly reduced in *Cxcr5*^−/−^ mice [34]. The involvement of sexual dimorphism in chronic pain mechanisms has been widely acknowledged [66, 67]. Our work also tested female mice and found that female *Cxcr5*^−/−^ mice showed similar improvement in mechanical allodynia compared with male mice. This result, thus, rules out the possible involvement of sexual dimorphism in CXCL13–CXCR5 signaling in CPIP model mice [34].

In this work, the upstream signaling related to CXCL13 overexpression was further explored and was found to be mediated by STAT3 signaling in spinal cord neurons, whereas CXCR5 was coupled to downstream NF-κB signaling and triggered proinflammatory cytokine IL-6 production from spinal cord neurons [34]. Previous work identified that IL-6 receptor (IL-6R) is predominantly expressed on microglia in spinal cord [68]. Thus, IL-6 produced by spinal neurons may in turn activate microglial IL-6R to promote microgliosis and initiate neuron–glia crosstalk that contributes to central sensitization and pain development as reported in previous work [68, 69]. Future work that can target IL-6 production specifically in spinal neurons is desirable to further support the role of neuronal IL-6 in mediating mechanical allodynia of CPIP model mice. Finally, in this work, targeted overexpression of CXCL13 in spinal cord dorsal horn neurons via a neuron-specific promoter is enough to trigger persistent mechanical allodynia in naive mice [34]. Therefore, this work reveals that neuronal CXCL13–CXCR5 signaling, via autocrine manner, contributes to mechanical and cold pain hypersensitivity in a mouse model of CRPS-I. Targeting the spinal CXCL13–CXCR5 pathway may present a novel therapeutic approach for CRPS-I management.

One particular interesting finding of this work is that CXCR5 expression was found to be exclusively upregulated in spinal neurons of CPIP model mice [34]. In contrast, two aforementioned studies revealed CXCR5 is predominantly upregulated in spinal astrocytes, instead of neurons, in spinal nerve ligation (SNL) model and PDN model mice, respectively [35, 48]. Therefore, it is likely that CXCR5 expression in spinal cord can be triggered in different types of cells under specific pathological pain conditions, which is worth of further investigation.

### CXCL13 in chronic constriction injury-induced neuropathic pain

Using an animal model of chronic constriction injury (CCI)-induced neuropathic pain model, one study identified a significant upregulation of CXCL13 and CXCR5 expression in the spinal cord of model animals [70]. The study further identified an important regulatory effect of transcription factor interferon regulatory factor 5 (IRF5) on CXCL13 expression in spinal cord. IRF5 is critically involved in both innate and adaptive immunity via conducting signals downstream of Toll-like receptors [71]. The authors found that IRF5 was overexpressed in spinal cord of CCI model rats, and it directly and specifically bound to the *Cxcl13* gene promoter, thus facilitating *Cxcl13* gene overexpression during chronic pain. *Irf5* gene knockdown reduced CXCL13 overexpression and alleviated neuropathic pain-like behavior in CCI model rats. It is, thus, proposed that upregulated IRF5 promotes CXCL13 overexpression in spinal cord, contributing to CCI-induced neuropathic pain [70]. CXCL13 may further be transported from the injured site to spinal cord or DRG via axonal transport, where it binds to the upregulated CXCR5 and mediates CCI-induced neuropathic pain [72]. In addition to peripheral sensory system and spinal cord, RNA-seq revealed *Cxcl13* gene expression was also significantly upregulated in the ACC of CCI model rats [73], a finding similar with results derived from SNL model animals [44]. This finding implicates a possible involvement of CXCL13 in mediating pain-related negative emotions of CCI model mice. Thus, further studies will be needed to validate this hypothesis.

### CXCL13 in orofacial neuropathic pain

Chronic neuropathic pain of orofacial region resulting from nerve trauma, compression, or demyelination is a debilitating pain condition and oftentimes refractory to treatments [74, 75]. Trigeminal ganglion (TG) is a crucial site for pain transmission and pain modulation from the peripheral to the central nervous system in the oral maxillofacial region. Partial infraorbital nerve ligation (pIONL) model is a commonly utilized animal model for the study of orofacial pain [76]. With the aid of this animal model, Zhang et al. reported a considerable increase in the expression of both CXCL13 and CXCR5 in TG of pIONL model mice [36]. Immunostaining revealed that CXCL13 and CXCR5 were both expressed in neurons of TG of pIONL model mice (Fig. 2, Table 1). The knockdown of the *Cxcl13* gene in TG before or after pIONL led to a notable decrease in mechanical hypersensitivity in model mice, indicating a critical role of CXCL13 in both development and maintenance phase of orofacial neuropathic pain [36]. Additionally, *Cxcr5*^−/−^ mice and *Cxcr5* gene knockdown mice showed significantly improved mechanical pain hypersensitivity after pIONL [36, 77]. The activation of CXCR5 results in ERK- and p38 MAPK-dependent proinflammatory cytokines (TNF and IL-1β) production in TG. Pharmacological blocking of ERK or p38 MAPK attenuates orofacial neuropathic pain in pIONL model animals [36, 77]. Thus, targeting CXCL13–CXCR5 and its downstream ERK or p38 MAPK signaling in TG may represent novel therapeutic approaches for orofacial neuropathic pain management.

### CXCL13 in complete Freund’s adjuvant-induced inflammatory pain

Complete Freund’s adjuvant (CFA) is a water-in-oil emulsion containing heat-killed mycobacteria [78]. The administration of CFA causes a series of inflammatory reactions, including macrophage activation and a massive infiltration of neutrophils [79]. Activated macrophages and neutrophils continually release large amounts of ROS and proinflammatory cytokines including TNF, IL-1β, and IL-6 that activate or sensitize peripheral nerve endings to trigger persistent pain [79]. Thus, the injection of CFA in animal footpad or joint represent well-established animal models for studying inflammatory pain. In a mouse model of CFA-induced inflammatory pain, Wu et al. identified a significant upregulation of the expression of CXCL13 as well as its receptor CXCR5 in ipsilateral DRG of model mice [37]. They further found that CXCL13 is broadly expressed in DRG neurons, whereas CXCR5 showed predominant expression in small-to-medium-sized DRG neurons after CFA treatment. *Cxcr5*^−/−^ mice exhibited a substantial improvement in both heat hyperalgesia and mechanical allodynia compared with wild-type mice after CFA treatment. To further investigate the mechanisms underlying how CXCR5 contributes to pain hypersensitivity of CFA model mice, the authors performed electrophysiology recording and found that the deletion of the *Cxcr5* gene reduced the enhancement in neuronal excitability of DRG neurons triggered by either CFA treatment or CXCL13 incubation, suggesting CXCR5’s involvement in modulating neuronal excitability [37]. Immunostaining uncovered a colocalization of Nav1.8, a sodium channel crucial for the induction of action potentials in nociceptive neurons, with CXCR5 in DRG neurons. Incubating neurons with CXCL13 dose dependently increased peak current density of Nav1.8 channel in DRG neurons via a CXCR5-dependent mechanism. It is known that Nav1.8 in DRG neurons can be phosphorylated by p38 MAPK, resulting in an increase in the current density in sensory neurons [80]. Therefore, the authors examined whether CFA treatment and CXCL13 can promote p38 MAPK activation in DRG neurons. They found that CFA and CXCL13 can both trigger p38 MAPK phosphorylation in DRG of WT mice but not in *Cxcr5*^−/−^ mice. Pharmacological blocking of p38 MAPK attenuates CXCL13-induced upregulation in Nav1.8 channel current density in DRG neurons, as well as mechanical and heat pain hypersensitivity, in CFA model mice [37]. Therefore, this study indicates that, upon tissue inflammation, DRG neuron-derived CXCL13 activates neuronal CXCR5 via an autocrine manner to enhance Nav1.8 channel activity in DRG neurons through p38-dependent mechanism, all of which contributes to peripheral sensitization and chronic inflammatory pain. Targeting peripheral CXCL13–CXCL5 signaling may be a potential method for ameliorating inflammatory pain.

### CXCL3 in rheumatoid arthritis (RA)

RA is a chronic and autoimmune disorder, inflaming and damaging the joints as well as other body parts, including the lung, heart, and eyes [81, 82]. The joint inflammation can cause chronic pain and ultimately lead to bone erosion, joint deformity, and even disability in patients [81]. The persistent joint pain is usually the most complained symptom among patients with RA [83].

TNF is considered as a key inflammatory cytokine involved in RA pathogenesis and progression [84]. TNF neutralizing biologics have been used in clinic to treat RA and reduce joint pain [84]. Studies reveal that TNF can activate tumor necrosis factor receptor (TNFR)1 on DRG neurons to potentiate TTX-resistant Na^+^ channels, thus promoting pain and peripheral sensitization [85, 86]. Additionally, TNF also activates TNFR2 on macrophages to facilitate macrophage accumulation in the DRG, resulting in neuroinflammation [85]. These two processes contribute to joint pain mechanisms in RA together. Accordingly, understanding how TNF is produced during RA is critical for identifying targets for RA inflammation and pain management.

CXCL13 has been found to be a potential biomarker for RA severity and is related with joint inflammation [87, 88]. In one recent study, it was found that CXCL13 incubation can trigger TNF production from the MH7A cell line that resembles human RA synovial fibroblasts [89]. CXCR5 expression was found to be significantly increased in synovial tissue of a mouse model of RA. CXCL13, via CXCR5, activates the downstream ERK and p38 signaling to promote TNF production in MH7A cells. The knockdown of the *Cxcl13* gene reduces TNF expression as well as joint inflammation in RA model mice [89]. It will be interesting to continue to examine whether joint pain is also ameliorated by *Cxcl13* gene knockdown. This work demonstrates an involvement of CXCL13–CXCR5 signaling in RA via promoting TNF production from synovial fibroblasts. Blocking CXCL13–CXCR5 signaling may provide therapeutic potentials to alleviate joint inflammation and pain in RA.

### CXCL13 in chronic postsurgical pain

Chronic postsurgical pain (CPSP) is among the most common complications for patients after a major surgery [90, 91]. It is estimated that 10–30% of patients complain prolonged pain 1 year after the surgery [91]. In a recent study, Yi et al. established a skin/muscle incision and retraction (SMIR) rat model to mimic CPSP and found that the expression of CXCL13 and CXCR5 was significantly increased in spinal cord tissues of model animals [92]. Intrathecal application of CXCL13 neutralizing antibody ameliorated SMIR-induced mechanical hyperalgesia and reduced nod-like receptor protein 3 (NLRP3) inflammasome activation, proinflammatory cytokine production (IL-1β and IL-18) as well as astrocyte overactivation in spinal cord of SMIR model rats. Furthermore, applying recombinant CXCL13 protein promoted the activation of NLRP3 inflammasome and the related inflammatory responses in primary rat astrocytes and increased glial fibrillary acidic protein (GFAP) expression in a dose-dependent manner. These effects were partially reversed by the application of INF39, a specific NLRP3 inflammasome inhibitor [93]. This study suggests that CXCL13–CXCR5 may promote neuroinflammation and chronic pain via activating NLRP3 inflammasome-dependent signaling in spinal cord of CPSP model animals. But it still remains to be further investigated how CXCR5 is exactly coupled with downstream NLRP3 inflammasome signaling under a CPSP condition.

### CXCL13 in bone cancer pain

Patients develop bone cancer pain (BCP) with primary bone cancer or secondary bone metastasis from distant sites, e.g., lung, prostate, and breast.[94]. BCP produces severe pain that dramatically affects the patients’ life quality. However, a majority of patients suffering from BCP usually receive insufficient pain management due to the relative limited analgesic effects and the adverse reactions of conventional therapeutics [95]. In terms of mechanisms, BCP is usually taken as a distinct type of pain with both overlapping and different characters of inflammatory and neuropathic pain [94, 96].

The contribution of spinal CXCL13–CXCR5 signaling to BCP has recently been reported [97]. BCP was established in rats via inoculating Walker 256 carcinoma cells in the tibia cavity. The expression of CXCL13 and CXCR5 was found to be increased in the spinal cord of model rats [97, 98]. The mechanical allodynia manifested by the BCP model rats were significantly alleviated by *Cxcr5* gene knockdown in spinal cord. Moreover, spinal injection of CXCL13 induced an increase in p-p38, p-ERK, and p-AKT expression in the spinal cord of BCP model rats, which was inhibited by spinal *Cxcr5* gene knockdown. This result indicates that CXCL13 may act upon CXCR5 to activate downstream p38, ERK, and AKT signaling to mediate BCP. However, the exact contribution of these downstream signaling pathway of CXCR5 to the pain hypersensitivity of BCP model animals still needs further investigation. Another study investigated the upstream regulatory mechanism of CXCL13 and found that lncRNA NONRATT009773.2 modulates CXCL13 expression by functioning as a microRNA sponge to absorb miR-708-5p in spinal cord of BCP model rats [98]. The upregulated CXCL13 may further induce neuroinflammation, including astrocyte overactivation and proinflammatory cytokine production in the spinal cord. In all, these studies suggest an involvement of spinal CXCL13–CXCR5 signaling in mediating BCP.

### CXCL13 in remifentanil-induced hyperalgesia

The chronic usage of opioids adversely induces pain, a phenomenon called opioid-induced hyperalgesia. Remifentanil is a short-acting opioid agonist with a lower risk of respiratory depression and delayed awakening after withdrawal. However, as an opioid, the usage of remifentanil is still limited by the occurrence of opioid-induced hyperalgesia [99]. Therefore, identifying mechanisms underlying remifentanil-induced hyperalgesia (RIH) is of clinical significance.

One study found that CXCL13–CXCR5 expression in the rat spinal dorsal horn was significantly increased after remifentanil intervention [100]. Intrathecal injection of CXCL13 neutralizing antibody dose dependently improved mechanical and thermal hyperalgesia in RIH model rats. To further explore how spinal CXCL13 contributes to RIH, the author focused on interleukin-17 receptor (IL-17)/interleukin-17 receptor A (IL-17RA) signaling. They found that the expression of IL-17 and its receptor IL-17RA in spinal dorsal horn of rats was also increased significantly following remifentanil intervention [100]. Spinal IL-17/IL-17RA signaling has been implicated in chronic pain [101]. IL-17/IL-17RA contributes to chemotherapy-induced peripheral neuropathy via mediating neuron-glial crosstalk and promoting neuron hyperexcitability in the spinal cord [102]. The authors found that CXCL13 neutralizing antibody inhibited the overexpression of IL-17/IL-17RA as well as the trafficking of GluN2B-containing NMDA receptor to the cell membrane. Furthermore, blocking spinal IL-17 reduced the trafficking of GluN2B-containing NMDA receptor from the cytosol to the membrane and reduced RIH in model rats [100]. Although the detailed mechanisms underlying how CXCL13 modulates IL-17/IL-17RA and GluN2B expression in RIH remains unknown, this finding demonstrates an important role of spinal CXCL13 in RIH via IL-17/IL-17RA-mediated GluN2B trafficking to the cell membrane. Therefore, spinal CXCL13 signaling may be a potential target for RIH management.

## CXCL13 signaling in neurological diseases

### CXCL13 in amyotrophic lateral sclerosis

Amyotrophic lateral sclerosis (ALS) is a fatal neurodegenerative disease characterized by progressive motor neuron degeneration that leads to muscle weakness, paralysis, respiratory insufficiency, and ultimate death [103]. Huge efforts have been devoted to identify potential therapeutic targets for slowing or preventing ALS’s progression. Recently, one study proposed the CXCL13–CXCR5 axis may be involved in ALS using both in vitro and in vivo experiments on ALS model mice and tissue samples from patients with ALS [104]. Using a mouse model that mimics ALS, the group found *Cxcl13* and *Cxcr5* gene expression was progressively upregulated in lumbar spinal cord of a fast progressing ALS model mice. Immunostaining revealed that CXCL13 was expressed by motor neurons and partially by microglial cells, whereas CXCR5 was exclusively expressed in motor neurons in the spinal cord of ALS model mice (Fig. 2, Table 1). CXCL13 was also detected to be progressively and remarkably released into cerebrospinal fluid (CSF) of the fast progressing ALS model mice. Moreover, the group identified a marked upregulation of CXCL13 associated with motor neurons and the surrounding axons as well as in efferent motor axons in the ventral portion of spinal cord of ALS model mice, indicating that CXCL13 was further transferred to the periphery axons after ALS. Intraventricular neutralization of CXCL13 accelerated motor functional impairment and reduced survival of ALS model mice. Pathology examination further revealed that motor neuron damage, astrocyte hyperplasia as well as denervation atrophy of hind limb skeletal muscles of ALS model mice were aggravated by intraventricular CXCL13 neutralization [104].

To further explore how CXCL13 contributes to ALS pathogenesis, the group established a primary coculture system containing motor neurons, astrocytes, and microglia obtained from spinal cord of ALS model mouse embryos. Silencing of *Cxcl13* expression exacerbated motor neuron loss and enhanced astrocytosis and microgliosis in the coculture system. Furthermore, the administration of recombinant CXCL13 protein to the coculture system under acute or chronic inflammatory condition protects motor neuron loss. The protective effect was abolished by CXCL13 neutralization. CXCL13 and CXCR5 were found to be upregulated in spinal motor neurons of patients with ALS, whereas CXCL13 levels in CSF of patients with ALS were lower than control subjects without neurological alterations [104]. The decreased CXCL13 level in CSF of patients with ALS is consistent with findings from the slow-progressing ALS model mice [104]. The authors interpreted that the lower CXCL13 levels in CSF might reflect motor neuron dysregulation in the spinal cord of both slow-progressing ALS model mice and patients with ALS. How this dysregulation in motor neuron occurs and how it affects CXCL13 release by motor neurons, however, still remain elusive. Thus, further studies will be needed to investigate this phenomenon in the slow-progressing ALS model mice and patients with ALS and its related mechanisms. Taken together, these data demonstrates an important role of CXCL13, expressed by spinal motor neurons, to reduce neuroinflammation and move alone motor axons to protect the degenerations in ALS model mice. The study implies that CXCL13 may be potentially used to ameliorate ALS. The study also indicates that the reduction in CXCL13 level in CSF of patients with ALS may possibly serve as a clinical indication for ALS progression (Table 2), although further clinical studies with more patients recruited are needed to validate this idea.

**Table 2:** Summary of the findings from clinical studies regarding CXCL13–CXCR5 expression dysregulation in pain or neurological diseases

Disease	Groups	Tissue	Results	Method	References
Amyotrophic lateral sclerosis (ALS)	Patients with ALS versus patients without ALS	Spinal cord motoneurons	CXCL13↑	Immunofluorescence	[104]
		CSF	CXCL13↓ (*p* < 0.05)	ELISA	
Rheumatoid arthritis (RA)	Patients with RA versus healthy controls	Plasma	CXCL13↑ (*p* < 0.0001)	ELISA	[88]
	Patients with RA after 6 months of treatment versus baseline	Plasma	CXCL13↓ (*p* < 0.0001)	ELISA	
Epilepsy	Patients with epilepsy versus nonepilepsy controls	Temporal neocortices	CXCL13↑ CXCR5↑ (*p* < 0.05)	qPCR Immunohistochemistry Western blotting	[108]
Multiple sclerosis (MS)	Patients with MS responding to 1 year teriflunomide treatment versus baseline	Serum	CXCL13↓ (*p* = 0.008)	Single molecule array	[110]
	Patients with MS versus non-neurologic disease controls	CSF	CXCL13↑ (*p* < 0.0001)	ELISA	[119]
LNB	Patients with LNB versus non-LNB controls	CSF	CXCL13↑ (*p* < 0.05)	Bio-Plex Pro human cytokine array	[111]
Anti-*N*-methyl-d-aspartate receptor encephalitis (anti-NMDAR encephalitis)	Patients with anti-NMDAR encephalitis versus non-anti-NMDAR encephalitis controls	CSF	CXCL13↑ (*p* < 0.0005)	ELISA	[112]

### CXCL13 signaling in ischemic stroke

A majority of strokes are caused by artery occlusion that results in brain ischemia. When the initial occlusion resolves, the reperfusion of blood with proinflammatory cytokines and immune cells further causes secondary injury that is usually more damaging than the initial occlusion [105]. There are no clinically approved drugs targeting the inflammatory component of ischemic stroke. Exploring how immune cells infiltrate to ischemic brain and their role in neuron death may provide new targets for ischemic stroke management.

Previous work shows that a group of CD4^+^ T cell can produce IL-21 and contributes to damage following transient middle cerebral artery occlusion (tMCAO) in mice and patients with ischemic stroke [106]. One recent study continued to explore the mechanisms leading to the recruitment of this specific group of CD4^+^ T cells [107]. They identified a group of IL-21-producing CXCR5^+^CD4^+^ICOS-1^+^ T follicular helper (T_FH_) cells infiltrated to the ischemic brain of model mice. The administration of CXCL13-neutralizing antibody reduced T_FH_ cells infiltration to the ischemic brain region and ameliorated ischemic injuries. Immunostaining revealed the presence of CD4^+^CXCR5^+^ T cells in ischemic brain regions and CXCL13^+^ vessels after ischemic injury in both model mice and human patients (Fig. 2, Table 1) [107]. The authors continued to look for the cellular distribution of IL-21 receptor, namely IL-21R, in the brain. They found that IL-21R was exclusively expressed in neurons, and its expression was upregulated during ischemic stroke in both mouse model and human patients (Fig. 2). This indicates a higher response can possibly be exerted by IL-21R-expressing neurons in response to IL-21 produced from infiltrated T_FH_ cells during ischemic stroke. In vitro experiments further showed IL-21 activated JAK/STAT pathway to trigger caspase-mediated apoptosis in hypoxic neurons [107]. Therefore, this study uncovers an important role of CXCL13 expressed on inflamed cerebral vessels in recruiting IL-21-producing T_FH_ cells via CXCR5 to produce neuroinflammation and subsequent neuronal death in ischemic stroke.

## Conclusions and future perspectives

Here, in this review, we summarize the most recent achievements in the study of CXCL13–CXCR5 signaling in the nervous system. We focus on the biological function, cellular expression, signaling transduction of the CXCL13–CXCR5 axis, as well as the cellular interactions this axis mediates during pathogenesis of chronic pain, and certain neurological diseases. We also outline the current strategies that can specifically target the CXCL13–CXCR5 axis in chronic pain or neurological diseases (Table 3). In addition to the documents summarized as above, there are some other studies that have identify dysregulation in CXCL13–CXCR5 in an animal model of perioperative neurocognitive disorder and epilepsy in both animal model and human patients, as well as multiple sclerosis (MS), lyme neuroborreliosis (LNB), and anti-*N*-methyl-d-aspartate receptor encephalitis (anti-NMDAR encephalitis) in human patients (Table 2) [108–112]. These studies in all may highlight an emerging importance of CXCL13 and CXCR5 as potential therapeutic targets or biomarkers for certain neurological diseases and brain inflammation.

**Table 3:** Strategies that can target or affect CXCL13–CXCR5 signaling for pain or neurological disease amelioration

Approaches	Experimental animal models	References
Recombinant CXCL13	PDN, RIH	[48, 100]
CXCL13 neutralizing antibody	SNL, CPIP, SMIR, RIH, ALS, tMCAO	[34, 40, 92, 100, 104, 107]
*Cxcr5*^*−/−*^	SNL, PDN, CPIP, pIONL, CFA	[34–37, 40, 48, 77]
*Cxcr5* knockdown	SNL, PDN, BCP, pIONL	[35, 36, 44, 48, 97]
*Cxcl13* knockdown	SNL, pIONL, RA	[35, 36, 40, 89]
*Cxcl13* overexpression	CPIP	[34]
miR-186-5p mimic LV-pre-mmu-miR-186 miR-186-5p inhibitor	SNL	[35]
miR-708-5p mimics LV-miR-708-5p miR-708-5p inhibitor	BCP	[98]

Although certain progress has been made in elucidating the contributions of CXCL13 signaling to chronic pain and certain neurological diseases, some research limitations still exist in this field that need further improvement in our view. First, the CXCL13–CXCR5 cellular expression patterns were exclusively derived from experiments with immunostaining. It is known that certain antibodies may lack validations of their specificities and the performance may not be consistent [113]. Therefore, the exact cellular distribution of CXCL13–CXCR5 in the nervous system needs to be further corroborated by means of some more reliable approaches, for example, in situ hybridization or the more advanced RNAscope technique. Second, it is apparent that CXCL13–CXCR5 exhibit expressions in both the immune and nervous systems. However, most of the current findings were derived from global *Cxcr5* knockout animals or regional gene knockdown approaches. Conditional knockout of *Cxcl13*/*Cxcr5* genes in specific types of cells or gene knockdown guided by tissue-specific promoter are still lacking. These techniques can help to unravel the precise involvement of CXCL13–CXCR5 signaling in specific cells or tissues in pathogenesis of chronic pain or neurological diseases. Third, there is emerging evidence demonstrating the presence of sexual dimorphism in mechanism of chronic pain [114–116]. It is known that chronic pain is usually more prevalent and more disabling in women than in men [117, 118]. However, most of the present findings indicating that CXCL13 signaling contributes to chronic pain are based upon experiments using male animals. Therefore, it is important to explore if CXCL13 signaling also contributes to chronic pain in female animals as well. If any human studies were performed to investigate the contribution of CXCL13–CXCR5 in chronic pain in the future, we suggest to discriminate between male and female human subjects. This can help to ascertain whether sexual dimorphism may exist in CXCL13 signaling in different chronic pain conditions.

Up to date, growing evidence has demonstrated the critical role of CXCL13–CXCR5 signaling in mediating neuroinflammation and the pathogenesis of chronic pain or certain neurological diseases. Strategies that can target CXCL13–CXCR5 signaling may provide new therapeutic options for these conditions.

## Data Availability

All data generated or analyzed during this study are included in this published article.

## Abbreviations

ACC: Anterior cingulate cortex
Akt: Protein kinase B
ALS: Amyotrophic lateral sclerosis
BCA-1: B cell attracting chemokine 1
BCP: Bone cancer pain
BLC: B lymphocyte chemoattractant
BLR1: Burkitt’s lymphoma receptor 1
CCI: Chronic constriction injury
CFA: Complete Freund’s adjuvant
CPIP: Chronic postischemic pain
CPSP: Chronic postsurgical pain
CRPS-I: Complex regional pain syndrome type I
CSF: Cerebrospinal fluid
CXCL13: Chemokine C–X–C motif ligand 13
CXCR5: C–X–C chemokine receptor type 5
DRG: Dorsal root ganglia
EPSCs: Excitatory postsynaptic currents
ERK: Extracellular signal-regulated kinase
GFAP: Glial fibrillary acidic protein
GPCR: G-protein-coupled receptor
IKK: Inhibitor of kappa B kinase
IL-17RA: Interleukin-17 receptor A
IL-21R: Interleukin-21 receptor
IRF5: Interferon regulatory factor 5
IκB: Inhibitor of NF-κB
JAK: Janus kinase
JNK: C-Jun N-terminal kinase
LNB: Lyme neuroborreliosis
MS: Multiple sclerosis
NF-κB: Nuclear factor-κB
NLRP3: Nod-like receptor protein 3
NMDAR: *N*-methyl-d-aspartate receptor
NSAIDs: Nonsteroidal anti-inflammatory drugs
p38 MAPK: p38 mitogen-activated protein kinase
PDN: Painful diabetic neuropathy
PI3K: Phosphatidylinositide 3-kinase
pIONL: Partial infraorbital nerve ligation
RA: Rheumatoid arthritis
RAS: Rat sarcoma
RIH: Remifentanil-induced hyperalgesia
SCDH: Spinal cord dorsal horn
SCVH: Spinal cord ventral horn
sEPSCs: Spontaneous excitatory postsynaptic currents
SMIR: Skin/muscle incision and retraction
SNL: Spinal nerve ligation
STAT3: Signal transducer and activator of transcription 3
T_FH_ cells: T follicular helper cells
TG: Trigeminal ganglion
tMCAO: Transient middle cerebral artery occlusion
TNFR: Tumor necrosis factor receptor
TNF: Tumor necrosis factor
ZNF382: Zinc finger protein 382

## References

[1] Yong RJ, Mullins PM, Bhattacharyya N. Prevalence of chronic pain among adults in the United States. Pain. 2022;163:e328–32. 33990113 10.1097/j.pain.0000000000002291

[2] Global burden of 369 diseases and injuries in 204 countries and territories, 1990–2019: a systematic analysis for the Global Burden of Disease Study 2019. Lancet. 2020; 396: 1204–22. 10.1016/S0140-6736(20)30925-9 PMC7567026 33069326

[3] Amodeo G, Magni G, Galimberti G, Riboldi B, Franchi S, Sacerdote P, et al. Neuroinflammation in osteoarthritis: From pain to mood disorders. Biochem Pharmacol. 2024;228:116182. 38556026 10.1016/j.bcp.2024.116182

[4] Dahlhamer J, Lucas J, Zelaya C, Nahin R, Mackey S, DeBar L, et al. Prevalence of Chronic Pain and High-Impact Chronic Pain Among Adults - United States, 2016. MMWR Morb Mortal Wkly Rep. 2018;67:1001–6. 30212442 10.15585/mmwr.mm6736a2 PMC6146950

[5] Racine M. Chronic pain and suicide risk: A comprehensive review. Prog Neuropsychopharmacol Biol Psychiatry. 2018;87:269–80. 28847525 10.1016/j.pnpbp.2017.08.020

[6] Degenhardt L, Grebely J, Stone J, Hickman M, Vickerman P, Marshall BDL, et al. Global patterns of opioid use and dependence: harms to populations, interventions, and future action. Lancet. 2019;394:1560–79. 31657732 10.1016/S0140-6736(19)32229-9 PMC7068135

[7] Berta T, Strong JA, Zhang JM, Ji RR. Targeting dorsal root ganglia and primary sensory neurons for the treatment of chronic pain: an update. Expert Opin Ther Targets. 2023;27:665–78. 37574713 10.1080/14728222.2023.2247563 PMC10530032

[8] Chen R, Yin C, Fang J, Liu B. The NLRP3 inflammasome: an emerging therapeutic target for chronic pain. J Neuroinflammation. 2021;18:84. 33785039 10.1186/s12974-021-02131-0 PMC8008529

[9] Salvador AF, de Lima KA, Kipnis J. Neuromodulation by the immune system: a focus on cytokines. Nat Rev Immunol. 2021;21:526–41. 33649606 10.1038/s41577-021-00508-z

[10] Chen O, Luo X, Ji RR. Macrophages and microglia in inflammation and neuroinflammation underlying different pain states. Med Rev. 2021;2023(3):381–407. 10.1515/mr-2023-0034 PMC10811354 38283253

[11] Ji RR, Nackley A, Huh Y, Terrando N, Maixner W. Neuroinflammation and Central Sensitization in Chronic and Widespread Pain. Anesthesiology. 2018;129:343–66. 29462012 10.1097/ALN.0000000000002130 PMC6051899

[12] Patani R, Hardingham GE, Liddelow SA. Functional roles of reactive astrocytes in neuroinflammation and neurodegeneration. Nat Rev Neurol. 2023;19:395–409. 37308616 10.1038/s41582-023-00822-1

[13] Li P, Yu Q, Nie H, Yin C, Liu B. IL-33/ST2 signaling in pain and itch: Cellular and molecular mechanisms and therapeutic potentials. Biomed Pharmacother. 2023;165: 115143. 37450998 10.1016/j.biopha.2023.115143

[14] Li Y, Yin C, Liu B, Nie H, Wang J, Zeng D, et al. Transcriptome profiling of long noncoding RNAs and mRNAs in spinal cord of a rat model of paclitaxel-induced peripheral neuropathy identifies potential mechanisms mediating neuroinflammation and pain. J Neuroinflammation. 2021;18:48. 33602238 10.1186/s12974-021-02098-y PMC7890637

[15] Xu R, Pan Y, Zheng K, Chen M, Yin C, Hu Q, et al. IL-33/ST2 induces macrophage-dependent ROS production and TRPA1 activation that mediate pain-like responses by skin incision in mice. Theranostics. 2024;14:5281–302. 39267790 10.7150/thno.97856 PMC11388077

[16] Yin C, Liu B, Li Y, Li X, Wang J, Chen R, et al. IL-33/ST2 induces neutrophil-dependent reactive oxygen species production and mediates gout pain. Theranostics. 2020;10:12189–203. 33204337 10.7150/thno.48028 PMC7667675

[17] Wei N, Guo Z, Qiu M, Ye R, Shao X, Liang Y, et al. Astrocyte Activation in the ACC Contributes to Comorbid Anxiety in Chronic Inflammatory Pain and Involves in The Excitation-Inhibition Imbalance. Mol Neurobiol. 2024;56:7. 10.1007/s12035-024-04027-5 38363535

[18] Wei H, Liu B, Yin C, Zeng D, Nie H, Li Y, et al. Electroacupuncture improves gout arthritis pain via attenuating ROS-mediated NLRP3 inflammasome overactivation. Chin Med. 2023;18:86. 37464384 10.1186/s13020-023-00800-1 PMC10355064

[19] Ji RR, Xu ZZ, Gao YJ. Emerging targets in neuroinflammation-driven chronic pain. Nat Rev Drug Discov. 2014;13:533–48. 24948120 10.1038/nrd4334 PMC4228377

[20] Yang Y, Pan Y, Liu B, Zhang Y, Yin C, Wang J, et al. Neutrophil-derived oxidative stress contributes to skin inflammation and scratching in a mouse model of allergic contact dermatitis via triggering pro-inflammatory cytokine and pruritogen production in skin. Biochem Pharmacol. 2024;223: 116163. 38522555 10.1016/j.bcp.2024.116163

[21] Li X, Yin C, Hu Q, Wang J, Nie H, Liu B, et al. Nrf2 Activation Mediates Antiallodynic Effect of Electroacupuncture on a Rat Model of Complex Regional Pain Syndrome Type-I through Reducing Local Oxidative Stress and Inflammation. Oxid Med Cell Longev. 2022;2022:8035109. 35498128 10.1155/2022/8035109 PMC9054487

[22] Du J, Cheng N, Deng Y, Xiang P, Liang J, Zhang Z, et al. Astrocyte senescence-like response related to peripheral nerve injury-induced neuropathic pain. Cell Mol Biol Lett. 2023;28:65. 37582709 10.1186/s11658-023-00474-5 PMC10428597

[23] Liu B, Chen R, Wang J, Li Y, Yin C, Tai Y, et al. Exploring neuronal mechanisms involved in the scratching behavior of a mouse model of allergic contact dermatitis by transcriptomics. Cell Mol Biol Lett. 2022;27:16. 35183104 10.1186/s11658-022-00316-w PMC8903649

[24] Jiang BC, Liu T, Gao YJ. Chemokines in chronic pain: cellular and molecular mechanisms and therapeutic potential. Pharmacol Ther. 2020;212: 107581. 32450191 10.1016/j.pharmthera.2020.107581

[25] Yin C, Liu B, Dong Z, Shi S, Peng C, Pan Y, et al. CXCL5 activates CXCR2 in nociceptive sensory neurons to drive joint pain and inflammation in experimental gouty arthritis. Nat Commun. 2024;15:3263. 38627393 10.1038/s41467-024-47640-7 PMC11021482

[26] Buchheit T, Huh Y, Maixner W, Cheng J, Ji RR. Neuroimmune modulation of pain and regenerative pain medicine. J Clin Invest. 2020;130:2164–76. 32250346 10.1172/JCI134439 PMC7190995

[27] Sallusto F, Baggiolini M. Chemokines and leukocyte traffic. Nat Immunol. 2008;9:949–52. 18711431 10.1038/ni.f.214

[28] Gunn MD, Ngo VN, Ansel KM, Ekland EH, Cyster JG, Williams LT. A B-cell-homing chemokine made in lymphoid follicles activates Burkitt’s lymphoma receptor-1. Nature. 1998;391:799–803. 9486651 10.1038/35876

[29] Legler DF, Loetscher M, Roos RS, Clark-Lewis I, Baggiolini M, Moser B. B cell-attracting chemokine 1, a human CXC chemokine expressed in lymphoid tissues, selectively attracts B lymphocytes via BLR1/CXCR5. J Exp Med. 1998;187:655–60. 9463416 10.1084/jem.187.4.655 PMC2212150

[30] Schaerli P, Willimann K, Lang AB, Lipp M, Loetscher P, Moser B. CXC chemokine receptor 5 expression defines follicular homing T cells with B cell helper function. J Exp Med. 2000;192:1553–62. 11104798 10.1084/jem.192.11.1553 PMC2193097

[31] Kazanietz MG, Durando M, Cooke M. CXCL13 and Its Receptor CXCR5 in Cancer: Inflammation, Immune Response, and Beyond. Front Endocrinol (Lausanne). 2019;10:471. 31354634 10.3389/fendo.2019.00471 PMC6639976

[32] León B, Ballesteros-Tato A, Browning JL, Dunn R, Randall TD, Lund FE. Regulation of T(H)2 development by CXCR5+ dendritic cells and lymphotoxin-expressing B cells. Nat Immunol. 2012;13:681–90. 22634865 10.1038/ni.2309 PMC3548431

[33] Pan Z, Zhu T, Liu Y, Zhang N. Role of the CXCL13–CXCR5 Axis in Autoimmune Diseases. Front Immunol. 2022;13: 850998. 35309354 10.3389/fimmu.2022.850998 PMC8931035

[34] Wang J, Yin C, Pan Y, Yang Y, Li W, Ni H, et al. CXCL13 contributes to chronic pain of a mouse model of CRPS-I via CXCR5-mediated NF-κB activation and pro-inflammatory cytokine production in spinal cord dorsal horn. J Neuroinflammation. 2023;20:109. 37158939 10.1186/s12974-023-02778-x PMC10165831

[35] Jiang BC, Cao DL, Zhang X, Zhang ZJ, He LN, Li CH, et al. CXCL13 drives spinal astrocyte activation and neuropathic pain via CXCR5. J Clin Invest. 2016;126:745–61. 26752644 10.1172/JCI81950 PMC4731172

[36] Zhang Q, Cao DL, Zhang ZJ, Jiang BC, Gao YJ. Chemokine CXCL13 mediates orofacial neuropathic pain via CXCR5/ERK pathway in the trigeminal ganglion of mice. J Neuroinflammation. 2016;13:183. 27401148 10.1186/s12974-016-0652-1 PMC4940825

[37] Wu XB, Cao DL, Zhang X, Jiang BC, Zhao LX, Qian B, et al. CXCL13–CXCR5 enhances sodium channel Nav1.8 current density via p38 MAP kinase in primary sensory neurons following inflammatory pain. Sci Rep. 2016;6:34836. 27708397 10.1038/srep34836 PMC5052602

[38] Zhao J, Huh Y, Bortsov A, Diatchenko L, Ji RR. Immunotherapies in chronic pain through modulation of neuroimmune interactions. Pharmacol Ther. 2023;248: 108476. 37307899 10.1016/j.pharmthera.2023.108476 PMC10527194

[39] Mauceri D. Role of Epigenetic Mechanisms in Chronic Pain. Cells. 2022;11:2613. 36010687 10.3390/cells11162613 PMC9406853

[40] Ma L, Yu L, Jiang BC, Wang J, Guo X, Huang Y, et al. ZNF382 controls mouse neuropathic pain via silencer-based epigenetic inhibition of Cxcl13 in DRG neurons. J Exp Med. 2021;218:45. 10.1084/jem.20210920 PMC8590274 34762123

[41] Cohen SP, Vase L, Hooten WM. Chronic pain: an update on burden, best practices, and new advances. Lancet. 2021;397:2082–97. 34062143 10.1016/S0140-6736(21)00393-7

[42] Bliss TV, Collingridge GL, Kaang BK, Zhuo M. Synaptic plasticity in the anterior cingulate cortex in acute and chronic pain. Nat Rev Neurosci. 2016;17:485–96. 27307118 10.1038/nrn.2016.68

[43] Chen T, Wang J, Wang YQ, Chu YX. Current understanding of the neural circuitry in the comorbidity of chronic pain and anxiety. Neural Plast. 2022;2022:4217593. 35211169 10.1155/2022/4217593 PMC8863453

[44] Wu XB, He LN, Jiang BC, Wang X, Lu Y, Gao YJ. Increased CXCL13 and CXCR5 in anterior cingulate cortex contributes to neuropathic pain-related conditioned place aversion. Neurosci Bull. 2019;35:613–23. 31041693 10.1007/s12264-019-00377-6 PMC6616612

[45] Feldman EL, Callaghan BC, Pop-Busui R, Zochodne DW, Wright DE, Bennett DL, et al. Diabetic neuropathy. Nat Rev Dis Primers. 2019;5:41. 31197153 10.1038/s41572-019-0092-1

[46] Qureshi Z, Ali MN, Khalid M. An insight into potential pharmacotherapeutic agents for painful diabetic neuropathy. J Diabetes Res. 2022;2022:9989272. 35127954 10.1155/2022/9989272 PMC8813291

[47] Dewanjee S, Das S, Das AK, Bhattacharjee N, Dihingia A, Dua TK, et al. Molecular mechanism of diabetic neuropathy and its pharmacotherapeutic targets. Eur J Pharmacol. 2018;833:472–523. 29966615 10.1016/j.ejphar.2018.06.034

[48] Liu S, Liu X, Xiong H, Wang W, Liu Y, Yin L, et al. CXCL13–CXCR5 signaling contributes to diabetes-induced tactile allodynia via activating pERK, pSTAT3, pAKT pathways and pro-inflammatory cytokines production in the spinal cord of male mice. Brain Behav Immun. 2019;80:711–24. 31100371 10.1016/j.bbi.2019.05.020

[49] Islam MS. Animal models of diabetic neuropathy: progress since 1960s. J Diabetes Res. 2013;2013: 149452. 23984428 10.1155/2013/149452 PMC3745837

[50] Gao YJ, Ji RR. c-Fos and pERK, which is a better marker for neuronal activation and central sensitization after noxious stimulation and tissue injury? Open Pain J. 2009;2:11–7. 19898681 10.2174/1876386300902010011 PMC2773551

[51] Xu JT, Tu HY, Xin WJ, Liu XG, Zhang GH, Zhai CH. Activation of phosphatidylinositol 3-kinase and protein kinase B/Akt in dorsal root ganglia and spinal cord contributes to the neuropathic pain induced by spinal nerve ligation in rats. Exp Neurol. 2007;206:269–79. 17628541 10.1016/j.expneurol.2007.05.029

[52] Wang J, Qiao Y, Yang RS, Zhang CK, Wu HH, Lin JJ, et al. The synergistic effect of treatment with triptolide and MK-801 in the rat neuropathic pain model. Mol Pain. 2017;13:1744806917746564. 29166839 10.1177/1744806917746564 PMC5734437

[53] Ott S, Maihöfner C. Signs and symptoms in 1,043 patients with complex regional pain syndrome. J Pain. 2018;19:599–611. 29409933 10.1016/j.jpain.2018.01.004

[54] Taylor SS, Noor N, Urits I, Paladini A, Sadhu MS, Gibb C, et al. Complex regional pain syndrome: a comprehensive review. Pain Ther. 2021;10:875–92. 34165690 10.1007/s40122-021-00279-4 PMC8586273

[55] Lloyd ECO, Dempsey B, Romero L. Complex regional pain syndrome. Am Fam Physician. 2021;104:49–55. 34264598

[56] Fassio A, Mantovani A, Gatti D, Rossini M, Viapiana O, Gavioli I, et al. Pharmacological treatment in adult patients with CRPS-I: a systematic review and meta-analysis of randomized controlled trials. Rheumatology (Oxford). 2022;61:3534–46. 35104332 10.1093/rheumatology/keac060

[57] Coderre TJ, Xanthos DN, Francis L, Bennett GJ. Chronic post-ischemia pain (CPIP): a novel animal model of complex regional pain syndrome-type I (CRPS-I; reflex sympathetic dystrophy) produced by prolonged hindpaw ischemia and reperfusion in the rat. Pain. 2004;112:94–105. 15494189 10.1016/j.pain.2004.08.001

[58] Hu Q, Zheng X, Chen R, Liu B, Tai Y, Shao X, et al. Chronic post-ischemia pain model for complex regional pain syndrome type-I in rats. J Vis Exp. 2020;89:7. 10.3791/60562 32065161

[59] Hu Q, Wang Q, Wang C, Tai Y, Liu B, Shao X, et al. TRPV1 channel contributes to the behavioral hypersensitivity in a rat model of complex regional pain syndrome type 1. Front Pharmacol. 2019;10:453. 31105572 10.3389/fphar.2019.00453 PMC6498414

[60] Chen R, Yin C, Hu Q, Liu B, Tai Y, Zheng X, et al. Expression profiling of spinal cord dorsal horn in a rat model of complex regional pain syndrome type-I uncovers potential mechanisms mediating pain and neuroinflammation responses. J Neuroinflammation. 2020;17:162. 32446302 10.1186/s12974-020-01834-0 PMC7245895

[61] Zhang Y, Chen R, Hu Q, Wang J, Nie H, Yin C, et al. Electroacupuncture ameliorates mechanical allodynia of a rat model of CRPS-I via suppressing NLRP3 inflammasome activation in spinal cord dorsal horn neurons. Front Cell Neurosci. 2022;16: 826777. 35693886 10.3389/fncel.2022.826777 PMC9174662

[62] Tang Y, Liu L, Xu D, Zhang W, Zhang Y, Zhou J, et al. Interaction between astrocytic colony stimulating factor and its receptor on microglia mediates central sensitization and behavioral hypersensitivity in chronic post ischemic pain model. Brain Behav Immun. 2018;68:248–60. 29080683 10.1016/j.bbi.2017.10.023

[63] Maria Frare J, Rodrigues P, Andrighetto Ruviaro N, Trevisan G. Chronic post-ischemic pain (CPIP) a model of complex regional pain syndrome (CRPS-I): Role of oxidative stress and inflammation. Biochem Pharmacol. 2024;229: 116506. 39182734 10.1016/j.bcp.2024.116506

[64] Yin C, Hu Q, Liu B, Tai Y, Zheng X, Li Y, et al. Transcriptome profiling of dorsal root ganglia in a rat model of complex regional pain syndrome type-I reveals potential mechanisms involved in pain. J Pain Res. 2019;12:1201–16. 31114302 10.2147/JPR.S188758 PMC6489655

[65] Sharma A, Agarwal S, Broatch J, Raja SN. A web-based cross-sectional epidemiological survey of complex regional pain syndrome. Reg Anesth Pain Med. 2009;34:110–5. 19282709 10.1097/AAP.0b013e3181958f90

[66] Sorge RE, Mapplebeck JC, Rosen S, Beggs S, Taves S, Alexander JK, et al. Different immune cells mediate mechanical pain hypersensitivity in male and female mice. Nat Neurosci. 2015;18:1081–3. 26120961 10.1038/nn.4053 PMC4772157

[67] Barcelon E, Chung S, Lee J, Lee SJ. Sexual Dimorphism in the Mechanism of Pain Central Sensitization. Cells. 2023;12:89. 10.3390/cells12162028 PMC10453375 37626838

[68] Hu Z, Deng N, Liu K, Zhou N, Sun Y, Zeng W. CNTF-STAT3-IL-6 Axis Mediates Neuroinflammatory Cascade across Schwann Cell-Neuron-Microglia. Cell Rep. 2020;31: 107657. 32433966 10.1016/j.celrep.2020.107657

[69] Lee JY, Park CS, Seo KJ, Kim IY, Han S, Youn I, et al. IL-6/JAK2/STAT3 axis mediates neuropathic pain by regulating astrocyte and microglia activation after spinal cord injury. Exp Neurol. 2023;370: 114576. 37863306 10.1016/j.expneurol.2023.114576

[70] Cao J, Hu C, Ding Z, Chen J, Liu S, Li Q. Mechanism of IRF5-regulated CXCL13–CXCR5 Signaling Axis in CCI-induced Neuropathic Pain in Rats. Curr Mol Med. 2023;7:940. 10.2174/1566524023666230825120836 37622691

[71] Roberts BK, Collado G, Barnes BJ. Role of interferon regulatory factor 5 (IRF5) in tumor progression: Prognostic and therapeutic potential. Biochim Biophys Acta Rev Cancer. 2024;1879: 189061. 38141865 10.1016/j.bbcan.2023.189061 PMC11977173

[72] Bu H, Jiao P, Fan X, Gao Y, Zhang L, Guo H. The role of botulinum toxin type A related axon transport in neuropathic pain induced by chronic constriction injury. Korean J Pain. 2022;35:391–402. 36175338 10.3344/kjp.2022.35.4.391 PMC9530680

[73] Zhang Y, Jiang S, Liao F, Huang Z, Yang X, Zou Y, et al. A transcriptomic analysis of neuropathic pain in the anterior cingulate cortex after nerve injury. Bioengineered. 2022;13:2058–75. 35030976 10.1080/21655979.2021.2021710 PMC8973654

[74] Ryan K, Crighton A. Trigeminal neuralgia and trigeminal neuropathic pain. Br Dent J. 2024;236:323–8. 38388612 10.1038/s41415-024-7068-6

[75] Romero-Reyes M, Arman S, Teruel A, Kumar S, Hawkins J, Akerman S. Pharmacological Management of Orofacial Pain. Drugs. 2023;83:1269–92. 37632671 10.1007/s40265-023-01927-z

[76] Xu M, Aita M, Chavkin C. Partial infraorbital nerve ligation as a model of trigeminal nerve injury in the mouse: behavioral, neural, and glial reactions. J Pain. 2008;9:1036–48. 18708302 10.1016/j.jpain.2008.06.006 PMC2632609

[77] Zhang Q, Zhu MD, Cao DL, Bai XQ, Gao YJ, Wu XB. Chemokine CXCL13 activates p38 MAPK in the trigeminal ganglion after infraorbital nerve injury. Inflammation. 2017;40:762–9. 28155010 10.1007/s10753-017-0520-x

[78] Apostólico Jde S, Lunardelli VA, Coirada FC, Boscardin SB, Rosa DS. Adjuvants: classification, modus operandi, and licensing. J Immunol Res. 2016;2016:1459394. 27274998 10.1155/2016/1459394 PMC4870346

[79] Noh ASM, Chuan TD, Khir NAM, Zin AAM, Ghazali AK, Long I, et al. Effects of different doses of complete Freund’s adjuvant on nociceptive behaviour and inflammatory parameters in polyarthritic rat model mimicking rheumatoid arthritis. PLoS ONE. 2021;16: e0260423. 34879087 10.1371/journal.pone.0260423 PMC8654228

[80] Hudmon A, Choi JS, Tyrrell L, Black JA, Rush AM, Waxman SG, et al. Phosphorylation of sodium channel Na(v)1.8 by p38 mitogen-activated protein kinase increases current density in dorsal root ganglion neurons. J Neurosci. 2008;28:3190–201. 18354022 10.1523/JNEUROSCI.4403-07.2008 PMC6670703

[81] Smolen JS, Aletaha D, Barton A, Burmester GR, Emery P, Firestein GS, et al. Rheumatoid arthritis. Nat Rev Dis Primers. 2018;4:18001. 29417936 10.1038/nrdp.2018.1

[82] Wu D, Luo Y, Li T, Zhao X, Lv T, Fang G, et al. Systemic complications of rheumatoid arthritis: Focus on pathogenesis and treatment. Front Immunol. 2022;13:1051082. 36618407 10.3389/fimmu.2022.1051082 PMC9817137

[83] Walsh DA, McWilliams DF. Mechanisms, impact and management of pain in rheumatoid arthritis. Nat Rev Rheumatol. 2014;10:581–92. 24861185 10.1038/nrrheum.2014.64

[84] Siegmund D, Wajant H. TNF and TNF receptors as therapeutic targets for rheumatic diseases and beyond. Nat Rev Rheumatol. 2023;19:576–91. 37542139 10.1038/s41584-023-01002-7

[85] Inglis JJ, Nissim A, Lees DM, Hunt SP, Chernajovsky Y, Kidd BL. The differential contribution of tumour necrosis factor to thermal and mechanical hyperalgesia during chronic inflammation. Arthritis Res Ther. 2005;7:R807–16. 15987482 10.1186/ar1743 PMC1175031

[86] Jin X, Gereau RWt. Acute p38-mediated modulation of tetrodotoxin-resistant sodium channels in mouse sensory neurons by tumor necrosis factor-alpha. J Neurosci. 2006; 26: 246–55. 10.1523/JNEUROSCI.3858-05.2006 PMC6674296 16399694

[87] Li Z, Chen Y, Zulipikaer M, Xu C, Fu J, Deng T, et al. Identification of PSMB9 and CXCL13 as Immune-related Diagnostic Markers for Rheumatoid Arthritis by Machine Learning. Curr Pharm Des. 2022;28:2842–54. 36045515 10.2174/1381612828666220831085608

[88] Greisen SR, Schelde KK, Rasmussen TK, Kragstrup TW, Stengaard-Pedersen K, Hetland ML, et al. CXCL13 predicts disease activity in early rheumatoid arthritis and could be an indicator of the therapeutic “window of opportunity.” Arthritis Res Ther. 2014;16:434. 25249397 10.1186/s13075-014-0434-z PMC4201737

[89] Achudhan D, Lai YL, Lin YY, Huang YL, Tsai CH, Ho TL, et al. CXCL13 promotes TNF-α synthesis in rheumatoid arthritis through activating ERK/p38 pathway and inhibiting miR-330-3p generation. Biochem Pharmacol. 2024;221: 116037. 38301965 10.1016/j.bcp.2024.116037

[90] Xu R, Wang J, Nie H, Zeng D, Yin C, Li Y, et al. Genome-Wide Expression Profiling by RNA-sequencing in spinal cord dorsal horn of a rat chronic postsurgical pain model to explore potential mechanisms involved in chronic pain. J Pain Res. 2022;15:985–1001. 35411184 10.2147/JPR.S358942 PMC8994637

[91] Bruce J, Quinlan J. Chronic Post Surgical Pain. Rev. Pain. 2011;5:23–9. 10.1177/204946371100500306 PMC4590073 26526062

[92] Yi H, Zhu B, Zheng C, Ying Z, Cheng M. CXCL13–CXCR5 promote chronic postsurgical pain and astrocyte activation in rats by targeting NLRP3. NeuroReport. 2024;35:406–12. 38526919 10.1097/WNR.0000000000002023

[93] Shi Y, Lv Q, Zheng M, Sun H, Shi F. NLRP3 inflammasome inhibitor INF39 attenuated NLRP3 assembly in macrophages. Int Immunopharmacol. 2021;92: 107358. 33508701 10.1016/j.intimp.2020.107358

[94] Zheng XQ, Wu YH, Huang JF, Wu AM. Neurophysiological mechanisms of cancer-induced bone pain. J Adv Res. 2022;35:117–27. 35003797 10.1016/j.jare.2021.06.006 PMC8721251

[95] Zajączkowska R, Kocot-Kępska M, Leppert W, Wordliczek J. Bone Pain in Cancer Patients: Mechanisms and Current Treatment. Int J Mol Sci. 2019;20:9. 10.3390/ijms20236047 PMC6928918 31801267

[96] Wang W, Zhou Y, Cai Y, Wang S, Shao F, Du J, et al. Phosphoproteomic Profiling of Rat’s Dorsal Root Ganglia Reveals mTOR as a potential target in bone cancer pain and electro-acupuncture’s analgesia. Front Pharmacol. 2021;12: 593043. 33995007 10.3389/fphar.2021.593043 PMC8117331

[97] Bu HL, Xia YZ, Liu PM, Guo HM, Yuan C, Fan XC, et al. The Roles of Chemokine CXCL13 in the Development of Bone Cancer Pain and the Regulation of Morphine Analgesia in Rats. Neuroscience. 2019;406:62–72. 30826523 10.1016/j.neuroscience.2019.02.025

[98] Chen J, Lu C, Wang X, Wang L, Chen J, Ji F. LncRNA NONRATT009773.2 promotes bone cancer pain progression through the miR-708–5p/CXCL13 axis. Eur J Neurosci. 2022;55:661–74. 35075718 10.1111/ejn.15607

[99] Santonocito C, Noto A, Crimi C, Sanfilippo F. Remifentanil-induced postoperative hyperalgesia: current perspectives on mechanisms and therapeutic strategies. Local Reg Anesth. 2018;11:15–23. 29670398 10.2147/LRA.S143618 PMC5898588

[100] Zhu M, Yuan ST, Yu WL, Jia LL, Sun Y. CXCL13 regulates the trafficking of GluN2B-containing NMDA receptor via IL-17 in the development of remifentanil-induced hyperalgesia in rats. Neurosci Lett. 2017;648:26–33. 28359934 10.1016/j.neulet.2017.03.044

[101] Gao SJ, Liu L, Li DY, Liu DQ, Zhang LQ, Wu JY, et al. Interleukin-17: a putative novel pharmacological target for pathological pain. Curr Neuropharmacol. 2024;22:204–16. 37581321 10.2174/1570159X21666230811142713 PMC10788884

[102] Luo H, Liu HZ, Zhang WW, Matsuda M, Lv N, Chen G, et al. Interleukin-17 regulates neuron-glial communications, synaptic transmission, and neuropathic pain after chemotherapy. Cell Rep. 2019;29:2384-97.e5. 31747607 10.1016/j.celrep.2019.10.085

[103] Brenner D, Freischmidt A. Update on genetics of amyotrophic lateral sclerosis. Curr Opin Neurol. 2022;35:672–7. 35942673 10.1097/WCO.0000000000001093

[104] Trolese MC, Mariani A, Terao M, de Paola M, Fabbrizio P, Sironi F, et al. CXCL13–CXCR5 signalling is pivotal to preserve motor neurons in amyotrophic lateral sclerosis. EBioMedicine. 2020;62: 103097. 33161233 10.1016/j.ebiom.2020.103097 PMC7670099

[105] Duan M, Xu Y, Li Y, Feng H, Chen Y. Targeting brain-peripheral immune responses for secondary brain injury after ischemic and hemorrhagic stroke. J Neuroinflammation. 2024;21:102. 38637850 10.1186/s12974-024-03101-y PMC11025216

[106] Clarkson BD, Ling C, Shi Y, Harris MG, Rayasam A, Sun D, et al. T cell-derived interleukin (IL)-21 promotes brain injury following stroke in mice. J Exp Med. 2014;211:595–604. 24616379 10.1084/jem.20131377 PMC3978271

[107] Rayasam A, Kijak JA, Kissel L, Choi YH, Kim T, Hsu M, et al. CXCL13 expressed on inflamed cerebral blood vessels recruit IL-21 producing T(FH) cells to damage neurons following stroke. J Neuroinflammation. 2022;19:125. 35624463 10.1186/s12974-022-02490-2 PMC9145182

[108] Li R, Ma L, Huang H, Ou S, Yuan J, Xu T, et al. Altered Expression of CXCL13 and CXCR5 in intractable temporal lobe epilepsy patients and pilocarpine-induced epileptic rats. Neurochem Res. 2017;42:526–40. 27873133 10.1007/s11064-016-2102-y

[109] Shen Y, Zhang Y, Chen L, Du J, Bao H, Xing Y, et al. Chemokine CXCL13 acts via CXCR5-ERK signaling in hippocampus to induce perioperative neurocognitive disorders in surgically treated mice. J Neuroinflammation. 2020;17:335. 33161894 10.1186/s12974-020-02013-x PMC7648984

[110] Fissolo N, Pappolla A, Rio J, Villar LM, Perez-Hoyos S, Sanchez A, et al. Serum Levels of CXCL13 Are Associated With Teriflunomide Response in Patients With Multiple Sclerosis. Neurol Neuroimmunol Neuroinflamm. 2023;10:1. 10.1212/NXI.0000000000200050 PMC9679885 36411079

[111] Pietikäinen A, Maksimow M, Kauko T, Hurme S, Salmi M, Hytönen J. Cerebrospinal fluid cytokines in Lyme neuroborreliosis. J Neuroinflammation. 2016;13:273. 27756335 10.1186/s12974-016-0745-x PMC5070144

[112] Liba Z, Kayserova J, Elisak M, Marusic P, Nohejlova H, Hanzalova J, et al. Anti-N-methyl-D-aspartate receptor encephalitis: the clinical course in light of the chemokine and cytokine levels in cerebrospinal fluid. J Neuroinflammation. 2016;13:55. 26941012 10.1186/s12974-016-0507-9 PMC4776396

[113] Voskuil JL. The challenges with the validation of research antibodies. F1000Res. 2017;6:161. 28357047 10.12688/f1000research.10851.1 PMC5333605

[114] Alarcón-Alarcón D, Cabañero D, de Andrés-López J, Nikolaeva-Koleva M, Giorgi S, Fernández-Ballester G, et al. TRPM8 contributes to sex dimorphism by promoting recovery of normal sensitivity in a mouse model of chronic migraine. Nat Commun. 2022;13:6304. 36272975 10.1038/s41467-022-33835-3 PMC9588003

[115] Luo X, Chen O, Wang Z, Bang S, Ji J, Lee SH, et al. IL-23/IL-17A/TRPV1 axis produces mechanical pain via macrophage-sensory neuron crosstalk in female mice. Neuron. 2021;109(2691–706): e5. 10.1016/j.neuron.2021.06.015 PMC8425601 34473953

[116] Luo X, Huh Y, Bang S, He Q, Zhang L, Matsuda M, et al. Macrophage toll-like receptor 9 contributes to chemotherapy-induced neuropathic pain in male mice. J Neurosci. 2019;39:6848–64. 31270160 10.1523/JNEUROSCI.3257-18.2019 PMC6733562

[117] Navratilova E, Fillingim RB, Porreca F. Sexual dimorphism in functional pain syndromes. Sci Transl Med. 2021;13:e7180. 10.1126/scitranslmed.abj7180 PMC12180430 34757805

[118] Umeda M, Kim Y. Gender differences in the prevalence of chronic pain and leisure time physical activity among US Adults: A NHANES Study. Int J Environ Res Public Health. 2019;16:988. 30893869 10.3390/ijerph16060988 PMC6466318

[119] Lundblad K, Zjukovskaja C, Larsson A, Cherif H, Kultima K, Burman J. CSF Concentrations of CXCL13 and sCD27 Before and After Autologous Hematopoietic Stem Cell Transplantation for Multiple Sclerosis. Neurol Neuroimmunol Neuroinflamm. 2023;10:5. 10.1212/NXI.0000000000200135 PMC10265403 37311645

